# Mechanistic Insight of Na/K-ATPase Signaling and HO-1 into Models of Obesity and Nonalcoholic Steatohepatitis

**DOI:** 10.3390/ijms21010087

**Published:** 2019-12-21

**Authors:** Rebecca Pratt, Hari Vishal Lakhani, Mishghan Zehra, Rutmann Desauguste, Sneha S. Pillai, Komal Sodhi

**Affiliations:** Departments of Surgery and Biomedical Sciences, Marshall University Joan C. Edwards School of Medicine, Huntington, WV 25701, USA; martin570@marshall.edu (R.P.); lakhani@marshall.edu (H.V.L.); humayun@marshall.edu (M.Z.); desauguste@live.marshall.edu (R.D.); pillais@marshall.edu (S.S.P.)

**Keywords:** oxidative stress, obesity, non-alcoholic steatohepatitis, Na/K-ATPase signaling, heme oxygenase 1

## Abstract

Obesity is a multifaceted pathophysiological condition that has been associated with lipid accumulation, adipocyte dysfunction, impaired mitochondrial biogenesis and an altered metabolic profile. Redox imbalance and excessive release of inflammatory mediators have been intricately linked in obesity-associated phenotypes. Hence, understanding the mechanisms of redox signaling pathways and molecular targets exacerbating oxidative stress is crucial in improving health outcomes. The activation of Na/K-ATPase/Src signaling, and its downstream pathways, by reactive oxygen species (ROS) has been recently implicated in obesity and subsequent nonalcoholic steatohepatitis (NASH), which causes further production of ROS creating an oxidant amplification loop. Apart from that, numerous studies have also characterized antioxidant properties of heme oxygenase 1 (HO-1), which is suppressed in an obese state. The induction of HO-1 restores cellular redox processes, which contributes to inhibition of the toxic milieu. The novelty of these independent mechanisms presents a unique opportunity to unravel their potential as molecular targets for redox regulation in obesity and NASH. The attenuation of oxidative stress, by understanding the underlying molecular mechanisms and associated mediators, with a targeted treatment modality may provide for improved therapeutic options to combat clinical disorders.

## 1. Introduction

Recent advances in understanding the pathophysiological progression of metabolic syndrome and, by extension, obesity and non-alcoholic steatohepatitis (NASH), has significantly contributed towards development of effective pharmacological and non-pharmacological strategies. However, clinical obesity remains a major worldwide epidemic, contributing to the increased morbidity and mortality due to its multifactorial origin. Oxidative stress remains a topic of great interest, as it is critically involved in the obesity-associated phenotype, the mechanisms of which has been thoroughly investigated by researchers. The dysregulated production of “offensive” adipocytokines in an obese state, along with excessive generation of reactive oxygen species (ROS) induces alterations in the metabolic profile that predisposes one to associated co-morbidities [1]. Although, the production of ROS has been implicated as an important part of adaptive responses in intracellular signaling and metabolic homeostasis [2,3], the decreased detoxifying ability of oxidant scavengers results in failure of preventing oxidative damage [4]. Hence, the interlinking relation between the systemic redox imbalance and release of inflammatory mediators creates an inflammatory milieu that significantly affects the regulation of metabolic pathways, consequences of which can lead to impaired physiological functions and the rise of derogatory pathological conditions [5]. Considering the elemental manifestation of oxidant stress in obesity, it is important to study novel molecular targets and implement innovative molecular strategies to curb oxidant injury. 

In light of understanding the modulation of redox mechanisms in a pathological setting, several dynamic cellular targets and processes have been proposed. The Na/K-ATPase oxidant amplification loop is one such profound signaling mechanism that has been causally associated with the exacerbation of oxidative stress in a diseased state [6,7]. The implication of this feed-forward mechanism is specifically of great interest, as the activation of Na/K-ATPase signaling is correlated with cellular ROS and cardiotonic steroids (CTS), which are both present in excess in the pathophysiological state of obesity. Once activated by the binding of ROS or CTS, the induction of conformational change and structural modulation of the Na/K-ATPase complex stimulates a signaling cascade [6]. The trigger of these signaling pathways activates mediators associated with the overproduction of ROS and inflammatory markers, hence creating a ROS amplification loop, exacerbating the already existing condition. The cumulative line of evidence suggests that the trigger of downstream signaling pathways mediated through activation of Na/K-ATPase signaling alters the adipocyte phenotype, induces mitochondrial dysfunction, modulates transcriptional and post-transcriptional regulation and, consequently, altering the systemic metabolic profile [8,9]. Hence, understanding the intersection of Na/K-ATPase oxidant amplification and redox imbalance in the obesity-associated phenotype can provide a viable target for disease intervention. 

Subsequently, previous studies have extensively characterized the antioxidant properties of the heme oxygenase (HO) system and its isoform, HO-1, which is a crucial stress responsive protein. A direct molecular effect of high oxidative stress has been implicated with suppression of HO-1, which is amendable to redox manipulations. The pathways associated with adipocyte dysfunction and altered metabolic homeostasis, cumulatively causing increased oxidative stress, induces downregulation of HO-1 [10,11]. Studies documenting the induction of HO-1 have highlighted its role in reducing visceral adiposity and ablating metabolic imbalance in obesity-associated phenotypes, and established the mechanistic basis for further investigating this antioxidant system [12,13,14,15]. The induction of HO-1 is applicable towards abatement of adipose tissue dysfunction, reduction of systemic inflammation, enhancement of adiponectin and restoration of metabolic balance [16]. The upregulation of this molecule and the intrinsic role of the HO-1 modality through which this anti-oxidant exerts its effect in a metabolically altered state, can provide insights to potential for therapeutic application of HO-1.

The novelty of the aforementioned mechanisms involved in the regulation of cellular redox present increasing importance in unraveling their potential as molecular targets of the obesity-associated phenotype and subsequent NASH. This review aims to independently uncover the mechanistic role of the Na/K-ATPase oxidant amplification loop, as well as the mechanisms associated with HO-1 regulation with increased oxidative stress in obesity and its associated comorbidity, NASH. This review will further assess the pharmacological and non-pharmacological strategies of manipulating localized redox signaling pathways mediated through the modulation of these mechanisms individually. 

## 2. Mechanistic Insights into Na/K-ATPase Oxidant Amplification Loop and Oxidative Stress

The Na/K-ATPase signaling cascade has been extensively studied due to its recently elucidated role as a scaffolding and signaling protein. Studies over the last two decades, since the emergence of the Xie Model of Na/K-ATPase signaling, have demonstrated that activation of this signaling pathway is pivotal in exacerbating oxidative stress in several disease models [8,9,17,18]. While several subunits of Na/K-ATPase have been dissected and known to be involved in its distinct ion pumping function, the α1 subunit of Na/K-ATPase signaling primarily causes its structural modulation leading to the activation of membrane-bound tyrosine kinase protein, Src [19,20]. The presence of excessive systemic ROS in a diseased condition induces carbonylation of the α1 subunit, which leads to the structural formation of the α1/Src molecular complex, subsequently leading to phosphorylation and activation of Src [19,21]. This critical binding of Src evidently has been shown to be induced by the N domain of the α1 subunit, which in turn transactivates the epithelial growth factor receptor (EGFR) [6]. Consequently, ROS-mediated activation of Na/K-ATPase/Src/EGFR signaling initiates the downstream signaling cascade, including the Ras-Raf-MEK-ERK pathway, resulting in amplified ROS with subsequent oxidative stress and the release of inflammatory mediators [22]. The cumulative line of evidence also suggests regulation of nicotinamide adenine dinucleotide phosphate (NADPH) oxidase derived superoxide generation through the activation of Src [23]. Hence, ROS-induced activation of Na/K-ATPase signaling and downstream pathways generates further ROS through a feed-forward mechanism and creates an Na/K-ATPase oxidant amplification loop, which has been shown to be critical in a diseased state. 

**Figure 1:**
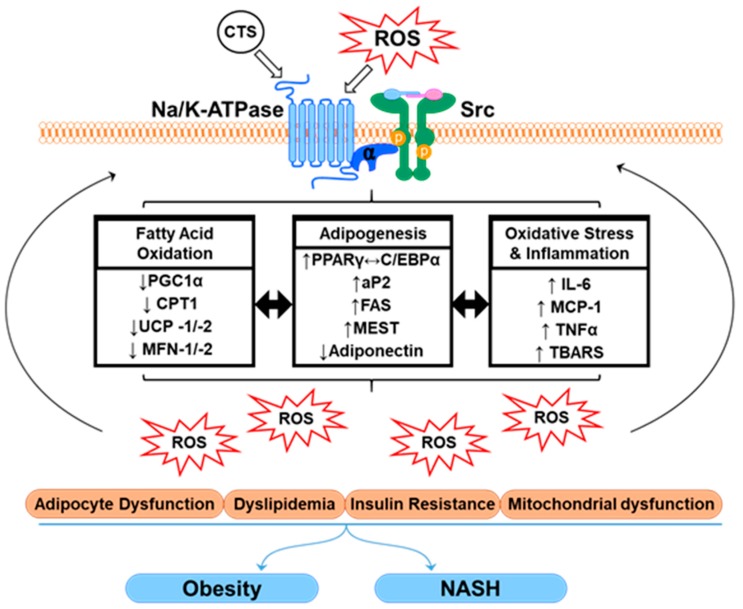
Schematic representation of the Na/K-ATPase oxidant amplification loop and associated mediators in inducing the obesity-associated phenotype. The activation of Na/K-ATPase α1 subunit through non-receptor-specific binding of ROS or receptor-specific binding of CTS in a diseased state, phosphorylates tyrosine kinase, Src. The formation of the α1/Src molecular complex activates downstream signaling pathways, causing alterations in regulation of markers associated with inflammation, oxidative stress, adipogenesis and mitochondrial fatty acid oxidation, which releases more ROS. This creates an oxidant amplification loop that further causes adipocyte dysfunction, dyslipidemia, insulin resistance and mitochondrial dysfunction, contributing to obesity and associated nonalcoholic steatohepatitis (NASH). Each arrow (shown as ↑ or ↓), represents the upregulation or downregulation of the respective genes or process.

While the cellular generation of ROS and the systemic redox imbalance evidently has been linked to the Na/K-ATPase/Src complex, the activation of this signaling mechanism can also be triggered by the release of CTS, like ouabain, which has been implicated in several diseased states [24,25,26,27,28]. Numerous studies have reported that Na/K-ATPase acts as a specific receptor for CTS. The binding of ouabain to the specific “receptor site” induces conformational changes in the molecular structure, which disrupts inactive Src kinase interactions, allowing for Src activation. Subsequently, this molecular activation of Src results in the protein interactions causing assembly and activation of the signaling cascade, including ERK cascades [19], the PLC/PKC pathway as well as inflammatory and pathways associated with cellular ROS generation, leading to oxidative stress [29]. The mechanisms operant in the activation of Na/K-ATPase oxidant amplification loop present increasing importance, providing therapeutic potential for the clinical conditions associated with oxidative stress and redox imbalance (Figure 1). 

## 3. Mechanistic Insights into HO-1 and Oxidative Stress

HO is the rate-limiting enzyme in the breakdown of heme in the body [30]. There are two major isoforms, HO-1 and HO-2, which are both expressed ubiquitously [31]. HO-1 is primarily recognized as a stress responsive protein, induced by various oxidative agents, and has the ability to degrade heme released from oxidant destabilized heme protein. This response is mediated by a variety of pro-oxidant and inflammatory stimuli, including oxidative stress, pharmacological compounds, transition metals, ultraviolet light, T helper cell cytokines, prostaglandins, lipopolysaccharide, dopamine, β-amyloid and the natural substrate, heme [30,32,33]. It cleaves pro-oxidant heme into carbon monoxide (CO), biliverdin (converted to bilirubin by biliverdin reductase) and free iron [33,34]. The ability of HO-1 to catabolize free heme and produce CO gives anti-inflammatory properties by up-regulation of interleukin-10 (IL-10) and interleukin-1 receptor antagonist (IL-1RA) expression [16,30,34,35]. Multiple studies have demonstrated the role of HO-1 in attenuating glucose-mediated cell growth arrest and reducing apoptosis in adipocytes, hepatic cells, endothelial cells and cardiac myocytes by formation of CO and activation of the p38 mitogen-activated protein kinase pathway [36,37]. Although the expression of HO-1 mediates several beneficial effects in the maintenance of metabolic homeostasis, HO-1 deficiency leads to sustained inflammation, nephropathy and tissue iron deposition. The altered expression of HO-1 also has been implicated in differentiation and cell growth [38]. In a rat fetal model, transduction of human HO-1 led to an increase in expression of insulin-like growth factor-1 receptor (IGF-1R) and vascular endothelial growth factor (VEGF), suggesting that HO-1 (or a byproduct of HO-1 such as CO or bilirubin) plays a critical role in signaling processes that enhance growth factor expression and activation [38,39]. 

**Figure 2:**
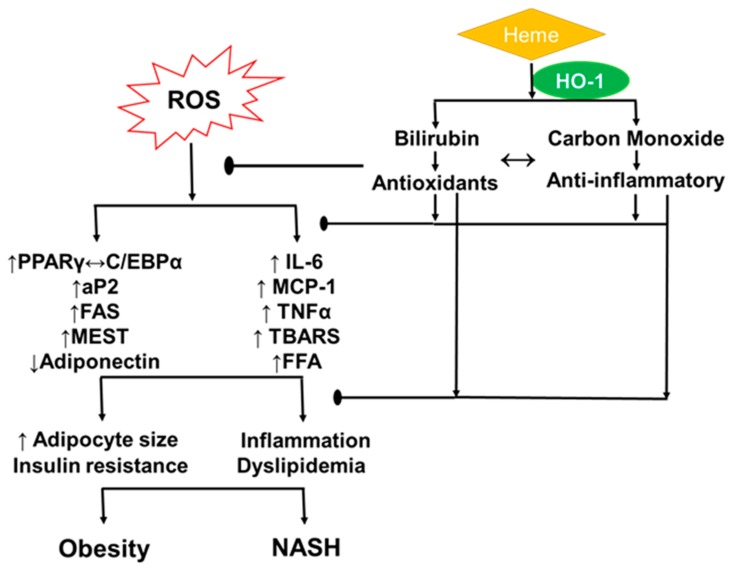
Schematic representation of the relationship between increased ROS, obesity, NASH and HO-1 intervention. Increased levels of ROS cause increases in inflammatory insult and adipogenic genes. This leads to increased adiposity, oxidative stress and dyslipidemia, which can exacerbate obesity and NASH. HO-1 breakdown of heme leads to production of bilirubin and carbon monoxide, which exert potent anti-oxidant and anti-inflammatory properties. Each arrow (shown as ↑ or ↓), represents the upregulation or downregulation of the respective genes or process.

Sirtuins have been implicated in glucose and lipid metabolism with an important role in various cellular metabolic pathways [40,41]. Recent studies have suggested a molecular interplay between HO-1 and sirtuin1 (SIRT1), demonstrating that upregulated expression of HO-1 activity can rescue SIRT1 expression, where the two form a “cytoprotective module” in a model of obesity [35]. Similarly, sirtuin4 (SIRT4) has been shown to be a regulator of metabolic enzymes and antioxidant defense mechanisms in mitochondria where it has a crucial role in regulating mitochondrial metabolism in response to exercise [42,43]. Previously published data have shown that SIRT4 is involved in a wide range of mitochondrial metabolic processes, including depressing insulin secretion in pancreatic cells, promoting lipid synthesis, regulating mitochondrial ATP, controlling apoptosis and regulating redox [43,44]. Cumulatively, the mechanisms operant in the alteration of HO-1 expression and the relationship between the mediators of oxidative stress presents increasing importance in the pathophysiological condition of obesity and subsequent NASH (Figure 2).

## 4. The Na/K-ATPase Oxidant Amplification Loop and HO-1: Implications for Obesity

Obesity and its associated morbidity and mortality have been on the rise, making it a global epidemic. Obesity is generally characterized by increased lipid accumulation and altered endocrine function of the adipose tissue along with dysfunctional adipogenesis being one of the major hallmarks of this clinical condition [45]. While numerous factors contribute to altered adipose tissue metabolism, redox imbalance has been largely implicated in the pathogenic mechanism for the maintenance of the obesity phenotype. Oxidative stress-induced adipogenesis and adipocyte phenotypic alterations increase adipogenic markers such as peroxisome proliferation factor-γ (PPAR-γ), fatty acid synthase (FAS) and mesoderm-specific transcript protein (Mest) along with pro-inflammatory cytokines, including tumor necrosis factor α (TNF-α), interleukin-6 (IL-6) and monocyte chemoattractant protein-1 (MCP-1) [8]. The excessive production of ROS and subsequent oxidative stress, in obesity, can also be partly attributed to the mitochondrial dysfunction and the altered state of mitochondrial biogenesis [9]. To this end, several studies have also demonstrated the important role of interferon-gamma (IFN-γ) in diet-induced obesity, which regulates systemic inflammation and insulin resistance in obesity [46,47]. In an obese state, the altered expression of IFN-γ promotes oxidized LDL production (oxLDL), causing adipose tissue macrophage dysfunction, and leading to increased oxidative stress, release of inflammatory cytokines and an altered adipocyte phenotype [47]. Therefore, the stimulation of ROS and subsequent generation of inflammatory mediators creates a vicious cycle, leading to an altered metabolic profile, increased lipid content and adipocyte dysfunction. 

The elucidation of the critical role of the Na/K-ATPase oxidant amplification loop in the generation of ROS, with subsequent oxidative stress and activation of downstream pathways, highlighted the importance of dissecting the mechanistic interplay of this signaling mechanism in obesity, given its interconnection with oxidative stress. This observation has led to equally innovative hypotheses over the past several years with extensive studies performed by the Xie/Shapiro laboratories revealing the underlying molecular mechanisms and phenotypic alterations in obesity caused by the Na/K-ATPase-mediated signaling pathways. The cumulative line of evidence demonstrated the ability of the Na/K-ATPase oxidant amplification loop in inducing adipocyte dysfunction in murine pre-adipocytes (3T3-L1) and further increasing adipogenesis [8]. Complementary in vivo studies in C57BL6 mice models with western diet (WD) interventions showed an increase in the adipogenic markers PPARγ, FAS and MEST along with decreased adiponectin expression in obesity [8]. Additionally, these in vivo obesity models exhibited a significant decrease in adipose tissue fat oxidation genes, markers of mitochondrial biogenesis and thermogenesis, including peroxisome proliferator-activated receptor gamma coactivator 1-alpha (PGC1α), carnitine palmitoyltransferase-1 (CPT1), uncoupling protein-1 and -2 (UCP -1 and -2), Sirt1 and mitofusin (MFN) -1 and -2 [8]. Subsequently, the diet-induced model of obesity altered the adipocyte phenotype, evidenced by the decreased number of adipocytes and increased adipocyte size [48]. To corroborate that these alterations were mediated by the oxidative stress-induced activation of the Na/K-ATPase oxidant amplification loop, these studies evidently reported the increase in protein carbonylation and thiobarbituric acid reactive substances (TBARS) expression, which are known markers of oxidative stress. Furthermore, the decreased expression of the α1 subunit with subsequent increase in the expression of c-Src and downstream phospho-ERK 1/2 in these in vivo studies accentuate the activation of Na/K-ATPase/Src signaling in the pathogenesis of obesity [8,48]. Studies also reported that this activation of Na/K-ATPase signaling in obesity led to the adipocyte-specific and systemic release of inflammatory mediators TNF-α, IL-6 and MCP-1, as well as insulin resistance, demonstrated by an increase in blood glucose and HOMA-IR levels [8,48]. Recently published data has reported similar findings suggesting the role of adipocyte-specific Na/K-ATPase signaling in obesity. Evidence shows that adipocyte dysfunction plays a causative role in the pathogenesis of obesity, including production of oxygen radicals through adipocyte mitochondria, mitochondrial lipogenesis and lipolysis [49]. Furthermore, adipocytes exposed to systemic oxidative stress upregulate the expression of inflammatory cytokines and macrophage chemoattractant molecules, contributing to more oxidative stress, aggravating the existing pathophysiological condition [50,51]. Hence, adipocytes create an ideal setting for the mechanistic action of Na/K-ATPase signaling and originating the effects of its downstream signaling pathways from adipocyte phenotype alteration. 

The cumulative line of evidence also suggests that the activation of NADPH oxidase in models of obesity leads to the subsequent activation of the renin–angiotensin system (RAS). The modulatory mediators of RAS, such as angiotensin II (AngII), increases cellular heme levels by peroxynitrite inactivation, produces excessive ROS and increases the release of inflammatory cytokines while decreasing expression of HO-1 and suppressing SIRT1. This redox imbalance leads to dyslipidemia and insulin resistance [40,41,52,53]. Heme increases lipid accumulation, promotes cell enlargement, induces over-expression of adipogenic genes, such as PPARɣ, C/EBP-α and adipocyte protein 2 (aP2), and downregulates adiponectin [32,34,54]. The increase in heme combined with increased ROS leads to adipocyte dysfunction by promoting inflammatory infiltration of macrophages and other inflammatory molecules, increases in circulating levels of glucose and a decrease in cytoprotective molecules, such as adiponectin and HO-1 [33,34,55]. Chronic oxidative stress leads to further adipocyte dysfunction, and can increase lipid accumulation [33,34]. The induction of HO-1 in disease models of obesity provide an antioxidant setting, which increases mitochondrial fusion and improves the secretory profile of adipocytes (i.e., increased adiponectin expression and decreased inflammatory cytokine release) [33,55]. HO-1 upregulation has also been linked to increased insulin sensitivity, improvement in phosphorylation of insulin receptors, and improved adipocyte function [35]. Previously published studies have shown the protective role of HO-1 in inhibiting the inflammatory effect of several mediators; inflammatory and oxidative transcription factors, such as nuclear factor kappa-light-chain-enhancer of activated B cells (NF-κβ) and c-Jun N-terminal kinase (JNK), which are primarily involved in inflammatory insult, stimulate inflammatory pathways, creating a feedback mechanism of inflammation [35,54,56]. Evidence from the literature suggests a potential interaction between HO-1 and adiponectin. Adiponectin is a protective adipokine and it has a significant role in insulin sensitivity and has a beneficial effect on triglycerides; adiponectin levels are inversely related to adiposity [48,57]. Reports have demonstrated that low adiponectin levels are associated with increased oxidative stress, and HO-1 expression is able to increase adiponectin levels [11,33]. Furthermore, adiposity has been shown to be attenuated by the HO-1-SIRT1 axis; alterations in adipocyte phenotype and inflammatory markers can be reversed when redox balance and SIRT1 are restored by HO-1 upregulation [16,35]. 

## 5. The Na/K-ATPase Oxidant Amplification Loop and HO-1: Implications for NASH

The development of obesity is often associated with co-morbidities, which includes manifestation of metabolic derangements leading to the phenotype of clinical NASH. The cluster of abnormalities associated with obesity, such as dyslipidemia and insulin resistance, have been known to be the causative factors of NASH. It is characterized by elevated hepatic adiposity, insulin resistance, increased free fatty acid (FFA) levels and increased inflammatory mediators [58]. Numerous studies have observed oxidative stress and inflammation in the underlying pathogenesis of this diseased phenotype. The systemic redox imbalance, along with inflammatory mediators and increased circulating levels of FFA in obesity predisposes one to NASH. This redox imbalance can generate highly toxic lipid peroxides that can induce hepatocellular injury and fibrogenesis [34,35,59,60]. This further contributes to altered hepatic mitochondrial fatty acid oxidation, triglycerides accumulation and steatosis [61]. In models of hepatocellular injury, increased ROS has also been linked with an impairment of insulin/insulin-like growth factor-1 (IGF-1) signaling and subsequent inhibition of the PI3K-Akt pathway [62,63]. This impairment is due to the effect of ROS on kinases downstream of the insulin receptor; the impairment of the IGF-1 pathway may be partly responsible for further damage to the liver in imbalanced redox states, making it a potential marker for insulin resistance and NASH associated with obesity [63,64]. The clinical manifestation and experimental models of diet-induced obesity with subsequent NASH has demonstrated large-scale production of ROS and chemoattractant cytokines through excessive hepatic lipid accumulation, further leading to the production of fibrogenic cytokines. 

The association of NASH with a redox imbalance exemplified the importance of investigating the role of Na/K-ATPase signaling and its downstream mechanisms as a target for this clinical condition. Recently published studies demonstrated the role of Na/K-ATPase signaling in aggravating oxidative stress and subsequent NASH using in vivo models of a WD diet. These studies extensively illustrated increased hepatic triglycerides levels, hepatic ALT, FFA, FAS and CD36 expression in mice fed a WD diet [9]. This lipid accumulation was confirmed by the histological findings of liver tissues, which exhibited a large number of vacuoles in the liver, significant inflammation and inflammatory cell infiltration into the liver. Similarly, studies have also demonstrated, by extension, a significant decrease in the expression of the long-chain acyl-CoA dehydrogenase (*LCAD*) gene associated with the first reaction of mitochondrial fatty acid oxidation and an important regulator for energy homeostasis along with significant decrease in PGC1α and CPT1 [9]. The oxidative stress induced by the activation of Na/K-ATPase signaling was also demonstrated to increase hepatic inflammatory markers, including F4/80, the marker of macrophage/kupffer cell infiltration, MCP-1 and IL-6. Further histological staining of liver tissues exhibited focal portal fibrosis, which provided evidence of steatohepatitis. These findings were also confirmed by the significantly increased expression of Type 1 collagen levels, which is a sensitive indicator of NASH, along with increased expression of hepatic fibronectin and matrix metalloproteinase9-and-13 (*MMP9* and *MMP13*), genes related to fibrogenesis. The manifestation of NASH in the diet-induced in vivo model was mediated by the oxidative stress and subsequent activation of Na/K-ATPase signaling, which was confirmed by the increase in protein carbonyls along with western blot analysis that showed activation of the Na/K-ATPase signaling downstream mediators Src and ERK 1/2 [9]. These observations are of potential interest and perhaps of great importance, considering that the ROS amplification in NASH is mediated by the Na/K-ATPase signaling pathway, and it may thus serve as a potential therapeutic target for NASH, which is characterized by oxidative stress, although much work is necessary to bridge the gap. 

Recent studies have hypothesized that HO-1 induction attenuates hepatic fibrosis by rescuing cellular SIRT1 and attenuating inflammation [35,65]. Evidence has linked increases in lipid peroxidation products with the release of cytochrome c from cells, which can lead to apoptosis, whereas increases in oxidative stress has been linked to hepatic tissue damage, causing hepatic fibrosis [31,35,66]. HO-1 plays a crucial role in anti-oxidative and cytoprotective defense mechanisms in the liver as well as the adipocyte. Bilirubin is capable of inhibiting lipid peroxidation and CO has potent anti-oxidant and anti-inflammatory properties that protect cells against oxidative stress [31,35,66]. HO-1 deficiency in mouse models and humans causes severe chronic hepatic inflammation, iron deposition and oxidative damage in the liver [31]. While the therapeutic potential and its mechanism of action are still largely unknown, HO-1 has been shown to attenuate experimental steatohepatitis, and the protective effects on cultured hepatocytes seems to indicate that HO-1 acts directly on hepatic tissue [31]. Upregulation of HO-1 gene expression leads to a reduction in hepatic lipid accumulation, improvement in insulin sensitivity and metabolic balance, and attenuates hepatic fibrosis [31,32,35]. 

## 6. Approaches to Targeted Inhibition of the Na/K-ATPase Oxidant Amplification Loop

The innovative breakthrough in the discovery of the scaffolding function of Na/K-ATPase signaling and its critical role in exacerbating diseased phenotype, led to the development of a synthetic peptide, pNaKtide, that specifically acts as an antagonist of this signaling mechanism. Although non-pharmacological, the effect of pNaKtide has been extensively demonstrated to inhibit the activation of Na/K-ATPase signaling, hence improving the diseased phenotype. The specific molecular composition and the sequencing for the development of pNaKtide has been detailed previously [28]; the exclusivity of which allows for its localization to the membrane component of the cell, which essentially inhibits formation of the Na/K-ATPase/Src complex and activation of downstream pathways. Studies elucidating the role of Na/K-ATPase signaling in obesity, specifically demonstrated an improved obesity-associated phenotype in vitro and in vivo by systemic administration of pNaKtide, which showed improved adipogenesis, with a marked reduction in the adipogenic markers, significant reduction in the overall body weight and reduced visceral and subcutaneous fat content [8]. These studies further corroborated their findings by demonstrating significant decrease in the oxidative stress and subsequent release of inflammatory cytokines, caused by the inhibited Na/K-ATPase α1 subunit expression, Src and downstream ERK 1/2 expression, with the systemic administration of pNaKtide. Similarly, the administration of pNaKtide in the murine models of steatohepatitis showed marked reduction in protein carbonylation along with significantly reduced inflammatory markers, lipid accumulation, improved insulin resistance and mitochondrial fatty acid oxidation [9]. Histological examination of liver tissues also showed the effectiveness of pNaKtide in attenuating hepatic fibrosis. This peptide has been demonstrated to effectively attenuate disease progression in atherosclerosis, evidenced by improved lipid accumulation in cardiac vasculature along with a reduction in the atherosclerotic plaques in the aorta. Subsequently, the administration of pNaKtide in a murine experimental uremic cardiomyopathy model showed reduced cardiac fibrosis and improved cardiac function, as demonstrated by the echocardiographic measurements [17]. Hence, the in vitro and in vivo models of disease progression mediated by the activation of the Na/K-ATPase oxidant amplification loop and its downstream signaling pathways, and the effective blockage of this signaling mechanism by pNaKtide, conclusively provides a strong evidence of its therapeutic efficacy. 

Recent advancements in the understanding of obesity progression led investigators to believe that adipocytes dysfunction plays a causative role in the pathogenesis of obesity and its associated comorbidities, rather than its previously well-established passive role. This led investigators to utilize a genetic approach using lentiviral vectors, a well-established, yet unique and promising strategy to specifically target adipocytes. Considering the effectiveness of systemic administration of pNaKtide in ameliorating the obesity phenotype, recent studies employed a lentiviral construct with NaKtide, a derivative of pNaKtide, which was driven by an adiponectin promoter to achieve its expression specifically in adipocytes and inhibit adipocyte Na/K-ATPase signaling [48]. Lentivirus with a NaKtide and adiponectin promoter (lenti-adipo-NaKtide) was injected intraperitoneally in mice fed a WD diet. The findings highlighted the importance of the Na/K-ATPase oxidant amplification loop within the adipocytes as well as the importance of adipocyte biology itself, which demonstrated an improved adipocyte phenotype, reduced overall body weight with reduction in fat content, lipid accumulation and attenuation of oxidative stress, as well as a subsequent reduction in inflammatory markers within the adipose tissue. While the adipocyte specific expression of NaKtide improved adipocyte mitochondrial biogenesis and adaptive thermogenesis, there was evidence of an improved systemic metabolic profile including reduced insulin resistance, systemic oxidative stress and plasma level of inflammatory cytokines [48]. These findings further advance our understandings of the basic underlying cellular mechanisms operant in obesity with the strong potential of NaKtide in clinical use, following the demonstration of its safe administration in humans. It appears not only that targeting the Na/K-ATPase oxidant amplification loop may be promising in terms of efficacy but may as well limit off-target effects.

While the use of non-pharmacological synthetic peptides, pNaKtide and the lentiviral approach using its derivative, NaKtide, seems to be highly effective in antagonizing Na/K-ATPase/Src signaling, several studies have also employed pharmacological strategies to inhibit Src. Several pharmacological inhibitors of the Src family kinase have been reported previously with high potency at low doses, including quinolinones (500nM), imminochromenes (2μM) and isothiazolones (4μM) [67]. Another pharmacological agent, PP2, a potent ATP-competitive inhibitor of Src, has been well studied with regards to Na/K-ATPase signaling and its antagonist, pNaKtide. Studies have demonstrated it effectiveness in inhibiting the basal Src activity in LLC-PK1 and cardiac myocytes, which was significantly greater than its counterpart, pNaKtide [28]. Furthermore, PP2 exhibited a similar IC50 on Src kinase as compared to pNaKtide. However, the specific modality of pNaKtide, which resides in the membranes, towards disrupting the formation of the Na/K-ATPase/Src receptor complex makes it unique, yet highly effective compared to PP2. While PP2 induces systemic inhibition of Src, pNaKtide specifically targets interaction of Na/K-ATPase-mediated Src activation, which limits the off-target effects. Similarly, other pharmacological inhibitors of Src offers limited specificity, thus limiting the clinical utility of these agents in attenuating oxidative stress-induced activation of the Na/K-ATPase/Src signaling cascade and the disease progression associated with it [68]. 

## 7. Approaches to Targeted Upregulation of HO-1

Cobalt protoporphyrin (CoPP) is an exogenous inducer of HO-1 that theoretically acts through the FoxO1 protein [54,56,69]. Current literature hypothesizes that FoxO1 negatively regulates adipogenesis by preventing transcription of PPARγ; decreased adipogenesis leads to a decrease in systemic oxidative stress and inflammatory cytokines [36,56]. Previous studies have shown that HO-1 induction by CoPP reduces visceral adiposity and attenuates metabolic imbalance, increases levels of phosphorylated AKT, AMPK, mTOR in adipocytes, improved oxidative stress, decreased release of inflammatory cytokines, decreased lipid accumulation and increased adiponectin levels [36,55,70]. Pharmacological inductions of HO-1 are not only limited to CoPP; hemin and epoxyeicosatrienoic acid (EET) have been implicated as pharmacological inducers of HO-1 [54,71].

Lentiviral vectors have been utilized as a gene targeting therapy in various models of disease, and HO-1 induction has been proven as successful [33,57]. Utilization of an adipocyte-specific (under the control of the aP2 promoter) lentiviral vector expressing HO-1 decreased adiposity and vascular dysfunction, improved metabolic parameters and attenuated serum levels of inflammatory cytokines in an in vivo model of diet-induced obesity [33]. Induction of HO-1 was accompanied by an increase in Wnt10b (pre-adipocyte marker) and a decrease in MEST (protein that correlates with adiposity and adipocyte size) [33]. Increased HO-1 expression also resulted in decreased levels of the genes involved in adipogenesis (PPARγ, C/EBPα and aP-2), which explains the decrease in adiposity. In addition, adiponectin, manufactured only in adipocytes, was increased after adipocyte-specific targeting of the HO-1 gene with a resultant improvement in adipocyte function [33]. 

Dietary antioxidants have been shown to increase HO-1 expression in vitro, and the potential use of this natural substances to regulate immune response should be carefully addressed [72,73]. A study performed by Scapagnini et. al. showed that low concentrations of caffeic acid phenethyl ester, or CAPE, and curcumin significantly increase HO-1 expression in astrocytes, and curcumin exerts a cytoprotective effect in endothelial cells, mediated by an increase in HO-1 gene expression and thus a decrease in oxidative stress [72,73]. It is believed that certain active components in medicinal plants induce the HO-1 gene; CAPE and curcumin are both known to inhibit NF-kB and cyclooxygenase activity [36,72,73]. They have also been shown to inhibit lipid peroxidation and cellular growth [72,73,74]. Increased physical activity has been shown to increase HO-1 induction and protect against oxidative stress in various models as well [75,76]. 

## 8. Conclusions

As the prevalence of obesity increases, it is paramount to note that the incidence of comorbidities, such as metabolic syndrome, NASH and cardiovascular disease, are going to increase as well. Obesity is associated with systemic oxidative stress, and it is suggested that impaired mitochondrial function and severe inflammation in the adipocyte underlie the pathogenesis of obesity [33,35,48]. Hypertriglyceridemia and hyperglycemia, both associated with increased adiposity, have been shown to increase plasma FFA, which can lead to increased ROS generation and oxidative stress; this imbalance and oxidative stress has been implicated in both NASH and cardiovascular diseases [34,35,48]. Understanding the role of ROS and oxidative stress in these disease states is a crucial step towards better therapeutic strategies. To this end, recent advancements in the understanding of both the Na/K-ATPase oxidant amplification loop and HO-1, provide a unique opportunity to better elucidate the redox mechanisms that modify inflammation and manipulate localized redox signaling pathways. It is highly possible that the mechanisms operant in both the Na/K-ATPase oxidant amplification loop and HO-1 may also involve other mediators (as yet unidentified) that directly modulate cellular oxidative and inflammatory responses. Although, the pharmacological and non-pharmacological interventions targeting these mechanisms have been demonstrated to be effective, it is possible that implementation of other strategies or targeting other pathways might prove to have better clinical outcomes. However, the success of these strategies will open up new avenues and approaches toward the antagonism of obesity and subsequent NASH, once we bridge the gap from mice to humans. 

## 9. Perspectives 

It has been shown that there is a central role for the Na/K-ATPase oxidant amplification loop in the pathogenesis of obesity as well as the commonly associated comorbidities. The specific mechanism is yet to be elucidated, but the role of HO-1 upregulation has also been characterized. Both of these signaling mechanisms present interesting therapeutic strategies for the treatment of obesity, NASH and cardiovascular diseases, while offering the possibility of limiting off-target effects. By increasing our understanding of the molecular biology and cellular mechanics involved we will not only gain an understanding of the exact sequence of events that precede these diseases, but also potentially have new biomarkers that act as a “warning system” for these diseases. Utilization of genetic therapies that specifically target the tissue of interest, as in the lentiviral gene therapy models, allows the opportunity for specific expression in target tissues, which limits off-target effects. Understanding the mechanics of a signaling pathway and its downstream targets would improve the understanding of disease progression. 

## Abbreviations

NASH	Nonalcoholic steatohepatitis
ROS	Reactive oxygen species
CTS	Cardiotonic steroids
HO	Heme oxygenase
EGFR	Epithelial growth factor receptor
NADPH	Nicotinamide adenine dinucleotide phosphate
CO	Carbon monoxide
IL-10	Interleukin-10
IL-1RA	Interleukin-1 receptor antagonist
SIRT1	Sirtuin1
FAS	Fatty acid synthase
PPAR-γ	Peroxisome proliferator-activated receptor gamma
CEBPα	CCAAT enhancer binding protein alpha
NFκB	Nuclear factor kappa light chain enhancer of activated B cells
UCP1	Uncoupling protein 1
UCP1	Uncoupling protein 2
MEST	Mesoderm specific transcript gene
MFN1	Mitofusin 1
MFN2	Mitofusin 2
MCP-1	Monocyte chemoattractant protein-1
TNFα	Tumor necrosis factor α
IL-6	Interleukin-6
WD	Western diet
PGC1α	Peroxisome proliferator-activated receptor gamma coactivator 1-alpha
CPT1	Carnitine palmitoyltransferase-1
Ang II	Angiotensin II
RAS	Renin-angiotensin system
aP2	Adipocyte protein 2
JNK	c-Jun N-terminal kinase
FFA	Free fatty acids
LCAD	Long-chain acyl-CoA dehydrogenase
MMP2	Matrix metalloproteinase 13
MMP9	Matrix metalloproteinase 9
EET	Epoxyeicosatrienoic acid
CAPE	Caffeic acid phenethyl ester
TBARS	Thiobarbituric acid reactive substances
IGF-1R	Insulin like growth factor-1 receptor
VEGF	Vascular endothelial growth factor
Sirt4	Sirtuin 4
IFN-y	Interferon γ
IGF-1	Insulin-like growth factor-1
GH	Growth hormone
